# Antisense inhibition of ATM gene enhances the radiosensitivity of head and neck squamous cell carcinoma in mice

**DOI:** 10.1186/1756-9966-27-56

**Published:** 2008-10-26

**Authors:** Jian Zou, Xiaoming Qiao, Huiping Ye, Yuqiong Yang, Xuelian Zheng, Houyu Zhao, Shixi Liu

**Affiliations:** 1 Department of Otolaryngology, West China Hospital, Sichuan University, Chengdu, 610041, PR China; 2 State Key Laboratory of Biotherapy, West China Hospital, Sichuan University, Chengdu, 610041, PR China; 3 Department of Oncology, West China Hospital, Sichuan University, Chengdu, 610041, PR China

## Abstract

**Background:**

Treatment failure after radiotherapy of head and neck squamous cell carcinoma (HNSCC) could be a significant problem. Our objective is to sensitize SCCVII cells to ionizing radiation *in vitro *and *in vivo *through inhibiting ATM expression using antisense oligodeoxynucleotides (AS-ODNs), and investigate the potential mechanism of radiosensitization.

**Methods:**

We designed and synthesized AS-ODNs that target ATM mRNA to reduce the ATM expression. The influence on the expression of ATM mRNA and protein in SCCVII cells were analysed by real-time quantitative PCR and western blotting respectively. Clonogenic survival assay was performed to detect the survival ability of SCCVII cells after irradiation, while flow cytometry used to analyse the cell cycle and apoptosis. The volume of solid tumors generated with SCCVII cells was measured, and cell apoptosis was analysed by TUNEL assay after irradiation.

**Results:**

The relative ATM mRNA and protein expression in SCCVII cells treated with ATM AS-ODNs were decreased to 25.7 ± 3.1% and 24.1 ± 2.8% of that in untreated cells respectively (*P *< 0.05). After irradiation, the survival fraction (SF) of cells treated with ATM AS-ODNs was lower than that of other groups at the same dose of radiation (*P *< 0.05), while the percentage of cells in G2/M phase decreased and apoptotic rate of cells increased(*P *< 0.05). The inhibition rate in SCCVII cells solid tumor exposed to X-ray alone was 23.2 ± 2.7%, while it was 56.1 ± 3.8% in the group which irradiated in combination with the treatment of ATM AS-ODNs (*P *< 0.05). The apoptotic index for the group irradiated in combination with ATM AS-ODNs injection was 19.6 ± 3.2, which was significantly higher than that of others (*P *< 0.05)

**Conclusion:**

Inhibition of ATM expression sensitized SCCVII cells to ionizing radiation *in vitro *and *in vivo*. The potential mechanism should be the defective G2/M cell cycle checkpoint control and enhanced radiation-induced apoptosis.

## Background

Despite advances in surgical treatments, radiotherapy is superior in its ability to preserve function and appearance in the treatment of head and neck squamous cell carcinoma (HNSCC). But some kinds of HNSCC are refractory to ionizing radiation, which results in the low effectiveness of radiotherapy alone[1,2]. SCCVII cell line, is a spontaneously arising head and neck squamous carcinoma cell line from syngeneic C3H/HeJ mice[3]. An oral cancer murine model using the SCCVII cell line shares characteristics such as initial locoregional tumor invasion, direct extension into the neck, and early cervical metastases with human head and neck tumors[4]. So SCCVII cell line could be a good object to study the biological behavior of HNSCC.

One strategy to improve the effectiveness of radiotherapy is augmenting of tumour radiosensitivity[5]. In the latter study, SCCVII cells were found to be resistant to ionizing radiation. The cytotoxicity of ionizing radiation is mainly mediated through the generation of DNA-double strand break (DSB) as evidenced by the pronounced radiosensitivity of cells and organisms defective in the machinery of DSB repair[6-8]. Thus, inhibition of DSB repair provides a mechanism to enhance the cytotoxicity of IR in tumour cells. The ataxia-telangiectasia mutated (ATM) protein kinase is a critical component in these pathways and integrates the cellular response to damage by phosphorylating key proteins involved in cell cycle regulation and DSB repair[9,10]. Lack of the normal ATM function in the inherited ataxia telangiectasia (AT) syndrome results in the profound hypersensitivity to ionizing radiation[11-13]. As mentioned elsewhere p53-wild-type cell lines with dysfunctional ATM, when irradiated, either show a lack of or delayed activation of p53, resulting in a defective G1/S cell-cycle checkpoint[14]. However, in p53 mutated cell lines, disruption of ATM resulted in defective G2/M checkpoint control, radio-resistant DNA synthesis, retarded cell proliferation and enhanced radiosensitivity[15,16]. Therefore, we manage to examine whether reduction of ATM expression after antisense oligodeoxynucleotides (AS-ODNs) treatment would result in enhanced radiosensitivity of p53-mutated SCCVII cells from C3H/He mice through the aberrant G2/M checkpoint.

## Methods

### Reagents

RPMI-1640 media and 10% heat-inactivated fetal bovine serum (FBS) were purchased from Gibco Company (Eggenstein, Germany). Lipofectamine 2000, Opti-MEM medium and Trizol kit were bought from Invitrogen Company(Carlsbad, CA, USA). SYBR ExScript RT-PCR Kit and SYBR Green Master Mix were purchased from Takara Biotechnology Company (Dalian, China). ATM monoclonal antibodies was bought from Santa Cruz Biotechnology (Santa Cruz, CA, USA), and β-actin monoclonal antibodies from Sigma (St Louis, MO, USA). BCIP/NBT alkaline phosphatase substrate kit IV was purchased from Vector laboratories (Burlingame, CA, USA). TUNEL apoptosis detection kit was bought from Roche Company (Shanghai, China)

### Cell lines and mice

SCCVII cell line was generously obtained from the laboratory of gene therapy at Johns Hopkins University. SCCVII cells were cultured in complete RPMI-1640 media containing 10% heat-inactivated FBS, 2 mM L-glutamine, 100 IU/mL penicillin, 100 μg/mL streptomycin. Cells were cultured as a monolayer at 37°C in a humidified atmosphere containing 5% CO2. Female C3H/He mice, aged 6–8 weeks, weighing 18–22 g, were obtained from Vital River Laboratories (Beijing, China) and were maintained in the animal facility at West China Medical School, Sichuan University in accordance with nation's related regulations and animal welfare requirements.

### Synthesis of oligodeoxynucleotides(ODNs) and selection of target sequences

A 25-mer AS-ODN which was previously reported to inhibit ATM expression in mouse cerebrovascular endothelial cells[17], and its associated controls, sense (Sen) and mismatch (Mis) ODNs, were synthesized by Shanghai Sangon Biological Engineering Technology & Services (Shangai, China). The sequences were as follows: AS, 5'-GTGCTAGACTCATGGTTTAAGATTT-3'; Sen, 5'-AAATCTTAAACCATGAGTCTAGCAC-3' and Mis, 5'-CCCCAGCAGCTCCCATTGGGCGTAA-3'. All the ODNs were chemically modified to phosphorothioate ODNs by substituting the oxygen molecules of the phosphate backbone with sulfur.

### Transfection of ODNs in SCCVII cells

SCCVII cells at a density of 5 × 10^4 ^cells/ml were plated for overnight incubation. Cells were maintained in RPMI-1640 medium supplemented with 10% FBS at 37°C and 5% CO2. After grew to 80%–90% fill, cells were replenished with incompleted RPMI-1640 medium, then treated with ATM AS-ODNs, ATM Sen-ODNs and Mis-ODNs. The procedures were as follows: 200 nM of ATM AS-ODNs, Sen-ODNs, Mis-ODNs and 2 mg/ml Lipofectamine 2000 were added to Opti-MEM medium separately, and incubated for 5 min at room temperature. Then liposome and ODNs were mixed together respectively and incubated at room temperature for 20 min. SCCVII cells were washed again with Opti-MEM medium before transfection. The liposome ODNs complexes were carefully plated on the cells, and incubated at 37°C, 5% CO2. After 6 hours transfected cells were washed twice with PBS, the medium was replaced with fresh RPMI-1640 medium supplemented with 10% FBS, cells were incubated at 37°C overnight. A second ODNs incubation was performed before cells were exposed to radiation.

### Real-time quantitative PCR analysis

Total RNAs were extracted from cultured SCCVII cells using Trizol reagent according to the manufacture's protocol. RNA concentration and purity were determined on a UV spectrophotometer (BioRad Inc., Hercules, CA, USA) by the 260 nm absorbance and 260–280 nm absorbance ratio, respectively. Synthesis of cDNA was conducted using SYBR ExScript RT-PCR Kit according to manufacturer's instructions. Real-time quantitative RT-PCR for the ATM mRNA was performed on an ABI PRISM 7300 Sequence Detection System (Applied Biosystems, Foster City, CA, USA) using SYBR Green Master Mix. For normalization the gene GAPDH was used. Final reaction volume is 25 μl. Cycling conditions were as follows: initial denaturation at 95°C for 10 s, followed by 40 cycles of 95°C for 5 s and 59°C for 31 s. Each measurement was performed in triplicate. The gene expression levels obtained were normalized by mRNA expression of GAPDH. The relative mRNA expression was then presented in relation to the untreated control group. All oligonucleotide primers were designed and synthesized by Sangon (Shanghai, China). The primer sequences are listed as follows: ATM, forward, 5'-CCAGGGGAAGATGATGAAGA-3' reverse 5'-CTACAATGAGCTGCGTGTGG-3'; GAPDH, forward,5'-CCTCAAGATTGTCAGCAAT-3' reverse, 5'-CCATCCACAGTCTTCTGAGT-3'.

### Western blot analysis

Total proteins extracted from SCCVII cells were separated on 10% or 15% SDS-polyacrylamide gels. Fifty micrograms each of the preparations were fractionated by 12.5% SDS-PAGE and transferred to nitrocellulose membrane (Millipore, Bedford, MA). The membrane was blocked with 3% milk powder in PBS at room temperature for 3 hours, washed with TBS (PBS containing 0.1% Tween-20) for 10 min three times, then incubated with the ATM monoclonal antibodies (1:1000 dilution) or β-actin monoclonal antibodies (1:2000 dilution) in TBS containing 1% milk powder at 4°C overnight. After three washes with TBS, the membrane was incubated with alkaline phosphatase-labeled anti-mouse IgG antibody in TBS containing 1% milk powder at room temperature for 3 hours and washed again with TBS three times. The membrane was briefly equilibrated with PBS and visualized with the BCIP/NBT alkaline phosphatases substrate kit IV. Reactive bands were scanned by Gel Doc 1000 (Bio-Rad). The experiment was repeated three times.

### Irradiation

ELEKTA Precise radiation system (Elekta, Sweden) was used to irradiate cells and solid tumor. X-ray irradiation was performed at room temperature at a dose rate of 200 cGy/min and equipped with an external 0.5-mm copper filter.

### Clonogenic survival assay

The SCCVII cells were seeded in triplicate at limiting dilutions in 6-well plates for about 24 hours in RPMI-1640 medium supplemented with 10% FBS until attached. Then the cells were transfected with ATM AS-ODNs, ATM Sen-ODNs and Mis-ODNs respectively. About 18 hours after transfection, they were irradiated with different doses of X-ray radiation(0, 2, 4, 6, and 8 Gy) respectively. The medium was replaced with a fresh one 24 hours after irradiation. After 7 days of incubation, the colonies were fixed with methanol, stained with 0.5% crystal violet in absolute ethanol and colonies with >50 cells were counted under dissection microscope. In each irradiation dose group, surviving fraction (SF) of cells was calculated as plating efficiency of the irradiated cells divided by the plating efficiency of the irradiated cells by that of the untreated control.

### Cell cycle and apoptosis analyzed by flow cytometry

After 48 hours exposed to 2 Gy radiation, cells were harvested, and centrifuged at 1500 rpm for 2 min. Then cells were washed with PBS twice, and fixed with 70% icy-cold ethanol at 4°C overnight. Cells were stained with PI at 4°C for 30 min. Cell cycle progression and apoptotic rate were analyzed by flow cytometry (Elite ESP, Beckman Coulter, USA).

### Animal experiment of radiosensitization

Female C3H/He mice were used to investigate the effect of ATM AS-ODNs on radiosensitivity of SCCVII cells solid tumor. All surgical procedures and care administered to the animals were in accordance with institutional guidelines. Animal surgeries and radiotherapy were performed under general anesthesia, 50 mg/kg ip injection of pentobarbital sodium. About 5 × 10^6 ^SCCVII cells were subcutaneously inoculated in submental space of the mice. Tumor growth rates were determined by measuring two orthogonal dimensional diameters of each tumor twice a week. Tumour volumes were estimated according to the formula V = π/6 × a2 × b, where a is the short axis, and b the long axis. When tumors reached an average volume of about 200 mm^3^, the tumor-bearing C3H/He mice were divided into four groups: (a) control group, no treatment; (b) ATM AS-ODNs group, tumors were treated with ATM AS-ODNs alone but not exposed to irradiation for each time; (c) irradiation group, tumors were exposed to X-ray of 2 Gy alone for each time; and (d) combination group, 2.5 mg/kg of ATM AS-ODNs was injected into the solid tumor the day before X-ray exposure, another dosage of ATM AS-ODNs was injected right before exposure to 2 Gy of X-ray for each time. The same treatment for each group were repeated 3 times (the interval time was 5 days). C3H/He mice were killed 3 weeks later. The ATM protein expression of the tumor in the different groups were ananlysed by western blot using the procedures described as above. The tumor inhibition rate was evaluated using the following formula: (1-average tumor volume of experimental group/average tumor volume of control group) × 100%.

### Terminal deoxynucleotidyltransferase-mediated dUTP-digoxigenin nick-end-labeling (TUNEL) assay

TUNEL staining of tumour sections was performed using an in situ apoptosis detection kit (Roche, Shanghai, China) according to the manufacture's protocol. The total number of apoptotic cells in 10 randomly selected fields was counted. The apoptotic index (AI) was calculated as the percentage of positive staining cells, namely AI = number of apoptotic cells × 100/total number of nucleated cells.

### Statistics

Results were expressed as mean ± standard deviation(SD). SPSS12.0 software package was used to perform statistical analysis. One-way ANOVA test was used to determine statistical difference between the experimental groups with others. Differences were considered statistically significant at *P *< 0.05.

## Results

### Expression of ATM in ATM AS-ODNs transfected SCCVII cells

**Figure 1:**
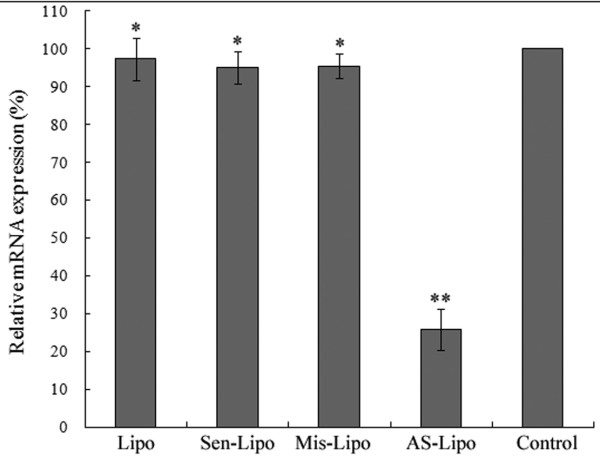
**Real-time quantitative PCR analysis of ATM mRNA expression**. Reduced expression of ATM mRNA in the presence of liposome formulations of ATM AS-ODNs(AS-Lipo) was observed. *P > 0.05, no significantly difference among liposome-treated group(Lipo), and Sen-ODNs (Sen-Lipo) treated group and Mis-ODNs (Mis-Lipo)treated group. **P < 0.05, compared with other groups.

**Figure 2:**
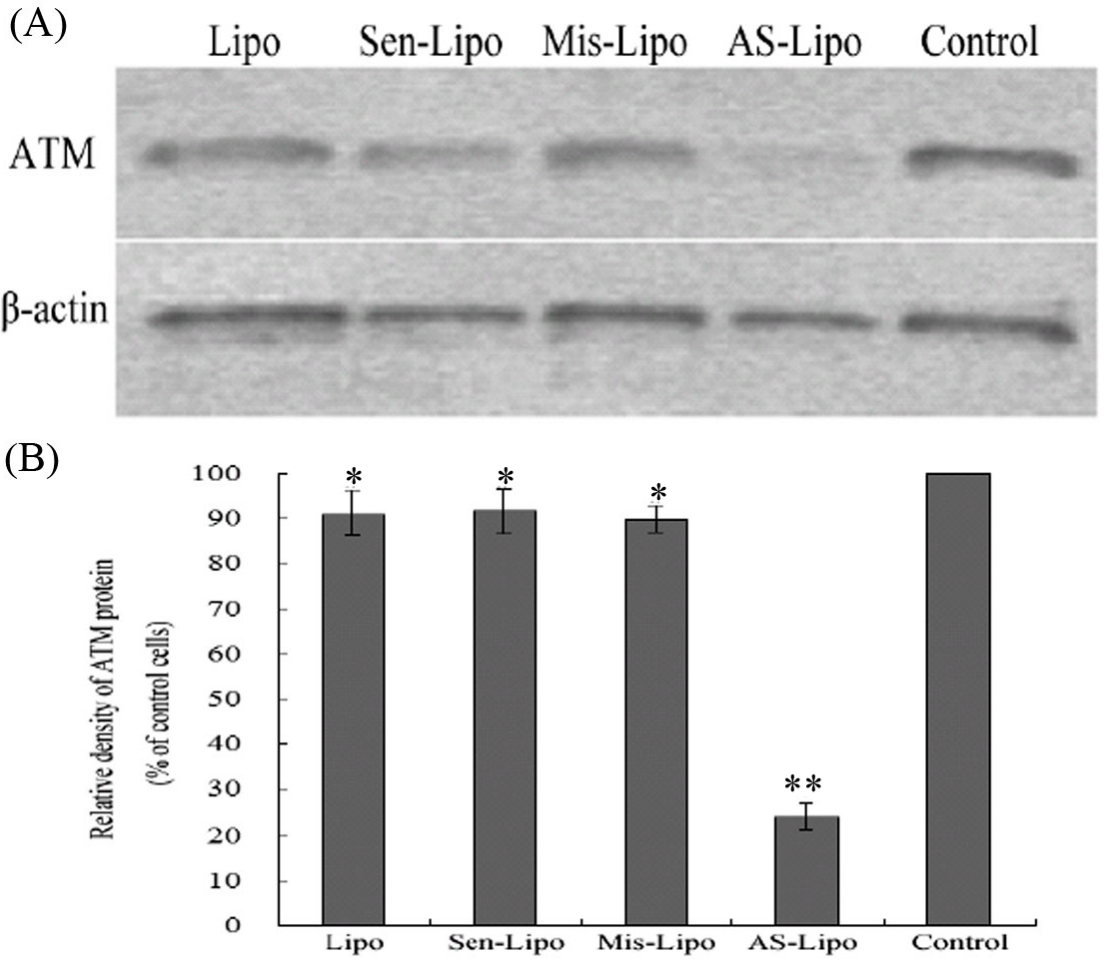
**Effect of ATM AS-ODNs on the expression of ATM protein *in vitro***. (A) Liposome formulations of ATM AS-ODNs significantly reduced the expression of ATM protein compared with other groups. (B) *P > 0.05, no significantly difference among liposome-treated group(Lipo), and Sen-ODNs (Sen-Lipo) treated group and Mis-ODNs (Mis-Lipo) treated group. **P < 0.05, compared with other groups.

We analyzed the expression of ATM in mRNA and protein level in SCCVII cells using real-time fluorescent quantitative PCR and western blot assay respectively. After 48 hours treatment, there were no significant difference among the group treated with liposome alone, the group treated with Sen-ODNs and the group treated with Mis-ODNs (*P *> 0.05; Figure. 1). However when incubating with liposome formulations of ATM AS-ODNs, the relative ATM mRNA expression was only about 25.7 ± 3.1% to the untreated SCCVII cells, which demonstrated a significantly reduced expression of ATM mRNA (*P *< 0.05; Figure. 1). As shown in Figure. 2, ATM protein expression was also significantly reduced by ATM AS-ODNs compared with Sen-ODNs and Mis-ODNs after 72 hours treatment (Figure. 2A). The relative ATM protein expression of SCCVII cells treated with ATM AS-ODNs was only about 24.1 ± 2.8% to the untreated cells (*P *< 0.05; Figure. 2B). But there was no significant difference among the group treated with liposome alone, the group treated with Sen-ODNs and the group treated with Mis-ODNs (*P *> 0.05; Figure. 2B).

### Effect of ATM AS-ODNs on clonogenic survival ability of SCCVII cells after irradiation

**Figure 3:**
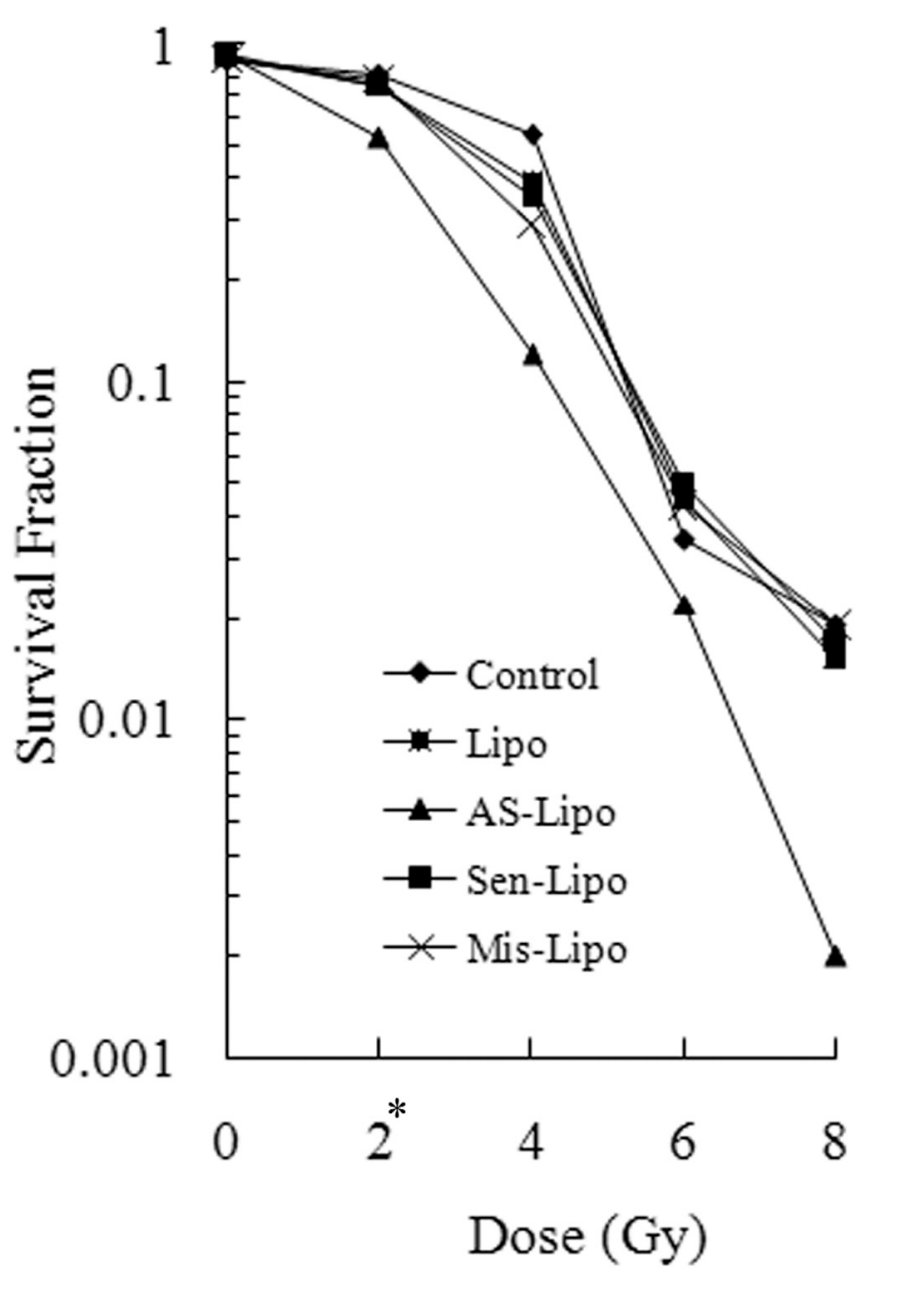
**Survival curves for SCCVII cells after irradiation**. Survival fractions at each dose point were normalized to untreated cells. * *P *< 0.05, The mean of SF2 in the cells transfected with ATM AS-ODNs was significantly lower than that of other cells.

Cellular response to ionizing radiation was evaluated by clonogenic survival assay. Compared with untreated cells or cells treated with control ODNs, cloning efficiency declined notably in cells which transfected with ATM AS-ODNs at the same dose of radiation (Figure. 3). The survival fraction after 2 Gy (SF2) reflect the cellular intrinsic radiosensitivity. The SF2 of cells transfected with ATM AS-ODNs was 53.3 ± 3.1%, definitely lower than that of other cells, which indicated a significant increase in the radiosensitivity (*P *< 0.05; Figure. 3). There were no obvious differences among the other groups about clonogenic survival ability (*P *> 0.05; Figure. 3).

### Effect of ATM AS-ODNs on apoptosis and cell cycle of SCCVII cells after irradiation *in vitro*

**Figure 4:**
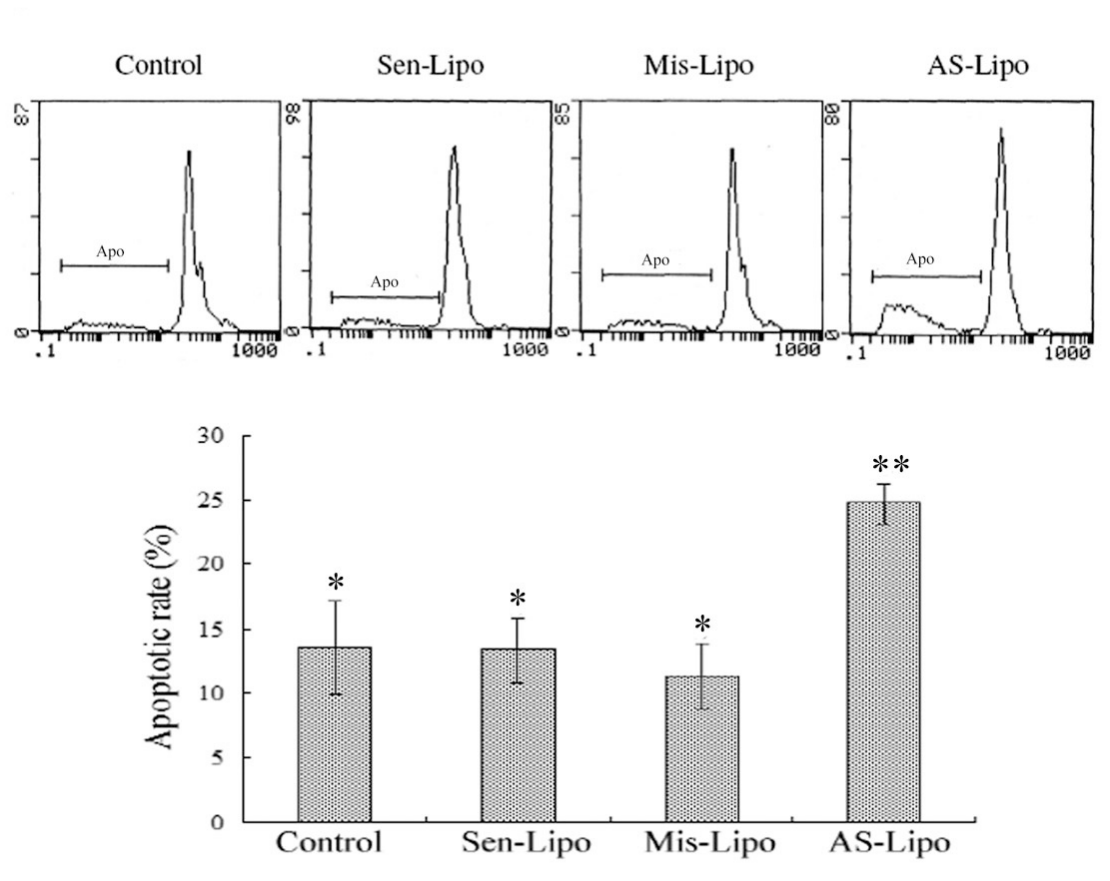
**The apoptotic rate of SCCVII cells after 2 Gy irradiation**. The apoptotic rate (Apo) in ATM AS-ODNs transfected cells was higher than that in Sen-ODNs and Mis-ODNs transfected cells after 2 Gy irradiation. * *P *> 0.05, no significant difference among these groups. ** *P *< 0.05, compared with other groups.

**Figure 5:**
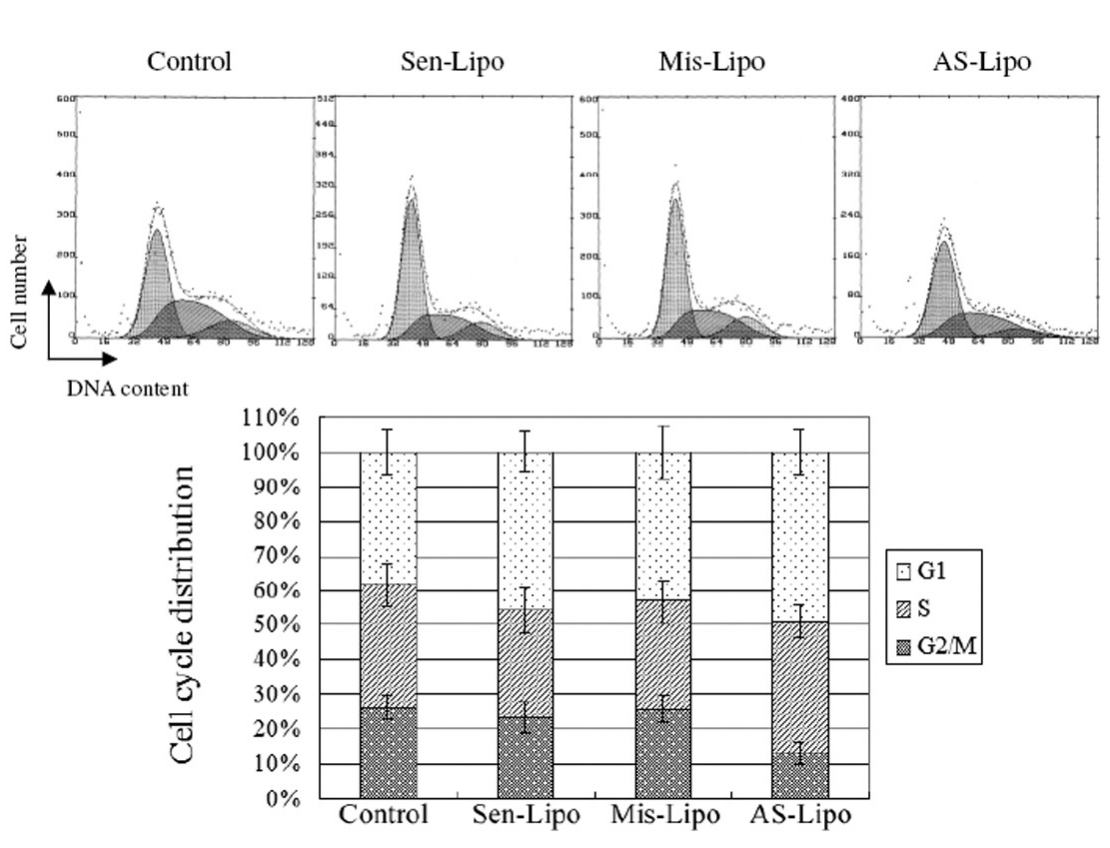
**Distribution of the cell cycle in SCCVII cells after 2 Gy irradiation**. The cell percentage of G2/M phase decreased dramatically in ATM AS-ODNs transfected cells at 48 hours after 2 Gy irradiation compared with that of other groups(*P *< 0.05).

After 2 Gy irradiation, the apoptotic rate in ATM AS-ODNs transfected cells was 24.7 ± 2.5%, which was higher than that in Sen-ODNs and Mis-ODNs transfected cells (*P *< 0.05; Figure. 4). It was also found that cell percentage of G2/M phase was decreased dramatically in ATM AS-ODNs transfected cells at 48 hours after 2 Gy irradiation compared with that of other groups(*P *< 0.05; Figure. 5).

### Inhibitory effect of ATM AS-ODNs on tumor growth *in vivo *after irradiation

**Figure 6:**
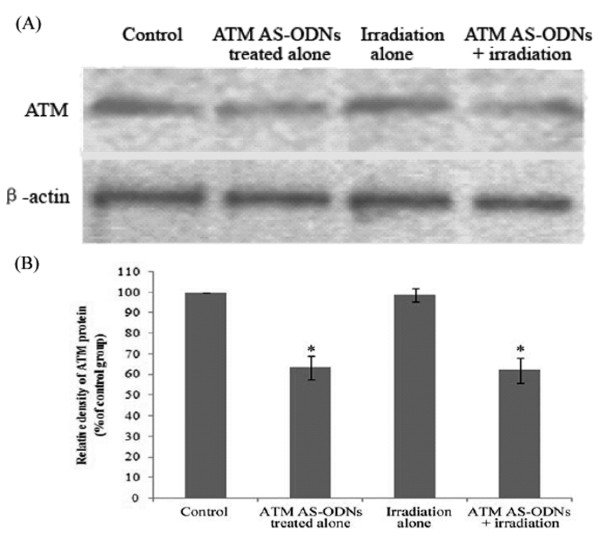
**Effect of ATM AS-ODNs on the ATM protein expression *in vivo***. (A) In the group treated with ATM AS-ODNs alone (ATM AS-ODNs treated alone) and the group irradiated in combination with ATM AS-ODNs (ATM AS-ODNs + irradiation), the expression of ATM protein were decreased. (B) * *P *< 0.05, compared with the untreated group and the group irradiated alone.

**Figure 7:**
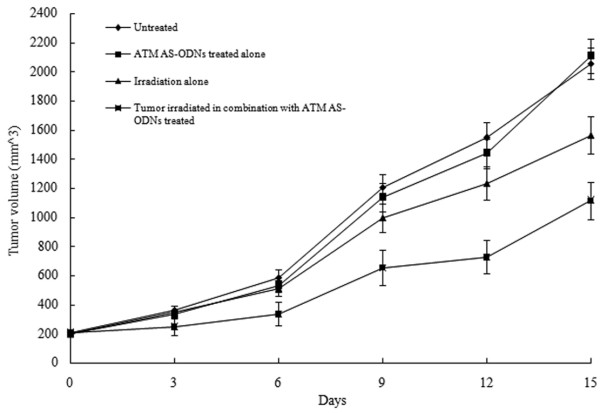
Tumor growth in ATM AS-ODNs treated SCCVII cells in C3H/He mice with or without irradiation.

In the group treated with ATM AS-ODNs alone and the group irradiated in combination with the treatment of ATM AS-ODNs, the relative ATM protein expression were only 63.4 ± 5.6% and 62.1 ± 6.1% to the untreated group respectively (*P *< 0.05; Figure. 6). Tumor growth of the mice in four groups were shown in Figure. 7. The inhibition rate in SCCVII cells solid tumor exposed to X-ray alone was 23.2 ± 2.7%, while it was 56.1 ± 3.8% in solid tumor irradiated in combination with the treatment of ATM AS-ODNs at the experimental endpoint(*P *< 0.05; Figure. 7).

### Enhancement of tumour apoptosis by irradiation combined with ATM AS-ODNs treatment *in vivo*

**Figure 8:**
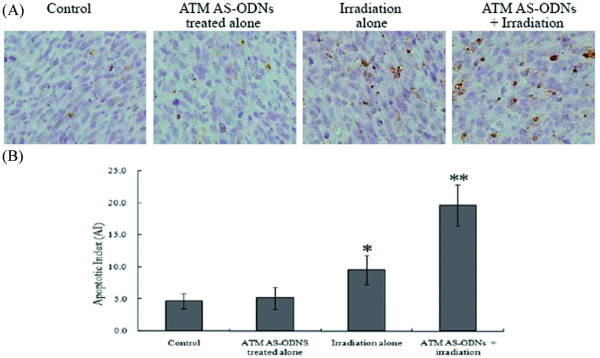
**The apoptosis of SCCVII cells *in vivo *after irradiation**. (A) Apoptotic cells are detected by TUNEL. The nuclei of apoptotic cells were stained brown as observed under light microscopy (magnification, × 400). (B) The treatment by irradiation in combination with ATM AS-ODNs injection enhanced the apoptotic rate of tumor cells. * *P *< 0.05, there was a significant difference in the AI of tumors treated with irradiation alone compared with the untreated group and the group treated with ATM AS-ODNs alone. ** *P *< 0.05, compared with the other groups.

Only small numbers of apoptotic cells were detected by TUNEL analysis in tumors treated with irradiation alone. In contrast with irradiation alone, tumor cell apoptosis was doubled following irradiation in combination with ATM AS-ODNs treatment (Figure. 8A). Accordingly, the AI for tumors from ATM AS-ODNs treated mice was 19.6 ± 3.2, significantly higher than that of the other groups (*P *< 0.05; Figure. 8B).

## Discussion

Damage to cellular DNA evokes a wide range of cellular responses that lead to activation of a variety of genes necessary for cellular survival, delay in cell-cycle progression, and induction of DNA repair[18-20]. ATM protein is a key mediator of the radioprotective machinery inducing a signaling network that is responsible for repair of radiation-induced damaged DNA and for cellular recovery and survival [21-23]. Rasheed had found that disruption of the ATM gene in mice resulted in exquisite sensitivity to low doses of ionizing radiation[24]. Yin demonstrated that treatment of mouse cerebrovascular endothelial cells with ATM AS-ODNs led to specific inhibition of ATM induction, and increased radiation-induced apoptosis *in vitro*[17]. Therefore we designed the experiment to test the hypothesis whether ATM AS-ODNs could inhibit the expression of ATM in SCCVII cells and furthermore increase the radiosensitivity by enhancing radiation-induced apoptosis *in vitro *and *in vivo*.

In the present study, we successfully transfected ATM AS-ODNs into SCCVII cells using liposome as delivery carrier, and detected the inhibitory expression of ATM at mRNA and protein level in SCCVII cells. We found that expression of ATM was dramatically reduced after cells were transfected with ATM AS-ODNs compared with that Sen-ODNs and Mis-ODNs treated groups, which indicated that the inhibition was specific for the ATM antisense sequence. Then we investigated whether the reduction of ATM expression resulted in radiosensitization in SCCVII cells. The results of clonogenic survival assay *in vitro *demonstrated that the cloning efficiency declined notably in cells which transfected with ATM AS-ODNs at the same dose of radiation (*P *< 0.05) compared with untreated cells or cells treated with control ODNs. While the SF2 of cells transfected with ATM AS-ODNs was 53.3 ± 3.1%, definitely lower than that of other cells, which means the increase of cell intrinsic radiosensitivity. Furthermore we investigated whether the increased radiosensitivity in SCCVII cells was due to the enhanced radiation-induced apoptosis and defective cell-cycle checkpoint. As we known, in p53 mutated cell lines, ATM mainly regulates the G2/M checkpoint to arrest cells in G2 phase at the time of irradiation where the radiation-induced DSBs can be repaired[15,16]. From flow cytometry, we found that the cells did not accumulate in the G2/M phase following irradiation in cells transfected with ATM AS-ODNs, which mean the reduced ATM expression resulted in the defective G2/M checkpoint control. Moreover, we found that radiation-induced apoptosis increased among the cells lack of ATM expression compared with those cells that have impact ATM expression.

In our study, we also investigated the effects of ATM AS-ODNs on the apoptotic responses to ionizing radiation *in viv*o. It was found that the tumors irradiated in combination with the treatment of ATM AS-ODNs were effective in controlling tumor growth and showed higher apoptotic rate. The inhibition rate in the tumors injected with ATM AS-ODNs before exposure to X-ray was 56.1 ± 3.8%, whereas it was 23.2 ± 2.7% in tumors exposed to radiation alone, and a significant difference was found between these two groups (*P *< 0.05). The results of TUNEL assay demonstrated that the apoptotic rate of the tumors irradiated in combination with the treatment of ATM AS-ODNs was obviously higher than that of control groups. The results of *in vivo *experiments indicated that the radiosensitivity of SCCVII cells solid tumors were enhanced by the treatment of ATM AS-ODNs and related with the increased radiation-induced apoptosis.

## Conclusion

We had demonstrated that the ATM AS-ODNs used in our study could specificly reduce the ATM expression and further result in an increased radiosensitivity in SCCVII cells *in vitro *and *in vivo*. The potential mechanism of radiosensitization related with reduced ATM expression should be the defective G2/M cell-cycle checkpoint control and enhanced radiation-induced apoptosis.

## Competing interests

The authors declare that they have no competing interests.

## Authors' contributions

XQ carried out cell culture, participated in the design of the study and performed the statistical analysis. HY carried out flow cytometry assay, participated in the animal experiment. YY participated in irradiation for cells and animals. XZ participated in the clonogenic survival assay. HZ carried out the TUNEL assays. SL conceived of the study, and participated in its design and coordination and helped to draft the manuscript. JZ designed the study, performed the rest of the experiments and wrote the manuscript. All authors read and approved the final manuscript.
